# Protocol for a randomized controlled trial of endoscopic ultrasound guided celiac plexus neurolysis (EUS-CPN) with vs without bupivacaine

**DOI:** 10.1186/s13063-023-07487-7

**Published:** 2023-09-08

**Authors:** Eslam Esmail, Sarto C Paquin, Anand V Sahai

**Affiliations:** 1 CHUM, Montreal, Canada; 2 Tropical Medicine Department, Tanta University, Tanta, Egypt

## Abstract

**Background:**

Pancreatic cancer is a devastating disease with less than 5% 5-year survival. Inoperable patients often present with pain. Randomized controlled trial have shown that endoscopic ultrasound-guided celiac plexus neurolysis (EUS-CPN) improves pain control. It is usually performed by injecting bupivacaine followed by absolute alcohol around the celiac axis.

**Study design:**

Single center, randomized, double blind controlled trial of EUS-CPN with and without bupivacaine in patients with inoperable malignancy (pancreatic or other) involving the celiac plexus. The study was approved by research ethics board with approval number of 2022-9969, 21.151 and registered on ClinicalTrials.gov (NCT04951804).

**Discussion:**

We hypothesize that bupivacaine is superfluous and may actually reduce pain control by diluting the neurolytic effect of alcohol. Bupivacaine is also potentially dangerous in that it may produce serious adverse events such as arrythmias and cardiac arrest if inadvertently injected intravascularly.

**Conclusion:**

This randomized trial is designed to assess whether bupivacaine is of any value during EUS-CPN.

**Supplementary Information:**

The online version contains supplementary material available at 10.1186/s13063-023-07487-7.

## Background

Pancreatic cancer is the most devastating of all cancers with a dismal survival rate. Pancreatic cancer is the 3rd most common cause of cancer-related deaths worldwide. The 5-year survival rate of all patients with pancreatic cancer is approximately 5%, and this figure has remained relatively stable over the past 25 years, since most patients present with inoperable disease [1, 2].

Seventy to 80% of patients with pancreatic cancer have abdominal pain at the time of diagnosis, which reduces quality of life and possibly survivability. Adequate pain control is therefore considered an essential component for the care of these patients. In the initial phase, the pain is visceral, but with disease progression, somatic pain may occur, especially due to the peri-pancreatic invasion of neural structures, such as the celiac plexus [3].

The effectiveness of CPN is well established. It is safe, produces significant pain reduction, significantly reduces narcotic requirements, and may even increase survivability [4–6]. Wyse et al. were the first to publish a randomized, sham-controlled trial demonstrating the efficacy of EUS-CPN for pain due to pancreatic cancer and authored the most recent published guidelines on the use of EUS-CPN [4, 7].

Currently, it is standard EUS-CPN practice to inject bupivacaine immediately before injecting absolute alcohol, to theoretically prevent pain during and after the procedure [7]. However, the true value of bupivacaine during neurolysis has never been studied. There are no data showing whether bupivacaine injection has any real influence on intra-procedural, immediate post-procedural, or long-term pain control. The injection of bupivacaine before the alcohol may have no effect, a synergistic effect, or an antagonistic effect, by diluting the alcohol and reducing its neurolytic capacity. Inadvertent intravascular injection of bupivacaine may also cause irreversible cardiac arrythmias and death [8]. In other words, in the worst-case scenario, the injection of bupivacaine may increase procedural risk, with questionable effect in terms of pain reduction.

In our institution, we stopped applying bupivacaine during EUS-CPN approximately 2 years ago, with no obvious difference intra- or post-procedure and a possible reduction in the need for repeat neurolysis—suggesting that neurolysis without bupivacaine may be more effective (unpublished observations).

Therefore, we designed a randomized clinical trial to determine if bupivacaine is of any value during EUS-CPN.

## Methods

### Objectives and design

The primary hypothesis for this study is that bupivacaine injection before neurolysis dilutes the neurolytic effect of alcohol and therefore decreases its analgesic effectiveness. We also believe that bupivacaine injection has no influence on intra- or immediate post-procedural pain.

The primary aim of study is to compare the effectiveness of EUS-CPN with and without bupivacaine. Secondary aims include a comparison of intra- and post-procedural pain, narcotic use, adverse events, and survival between the studied groups.

This is a single-center, randomized, double-blinded, parallel groups, non-inferiority clinical trial to be performed at the endoscopy unit of the Centre Hospitalier de l’Université de Montréal (CHUM) in Québec, Canada.

The SPIRIT reporting guidelines have been used in protocol formulation [9].

### Study population

All patients referred for EUS for suspected pancreatic malignancy or for neurolysis for pain due to proven pancreatic malignancy are eligible. The study will be presented to them by a single research assistant, who will also obtain study consent before the procedure. Patients will be randomized if they meet all inclusion criteria and have no exclusion criteria presented in Table 1.

**Table 1:** Inclusion and exclusion criteria for the study

**Inclusion criteria**	1. Malignant-appearing pancreatic mass, or proven pancreatic cancer involving the pancreatic genu, body, or tail on imaging done before the endosonography 2. Any level of abdominal or back pain considered to be potentially related to the mass: a. New onset pain (< 3 months) b. Constant c. Centrally located d. With or without irradiation to the back e. No obvious other source of pain 3. No possibility of immediate surgical management. (Based on EUS evidence of venous involvement ≤ 1 cm from portal confluence and/or arterial involvement of the celiac axis or the splenic artery ≤ 1 cm from the bifurcation of the celiac axis, and/or evidence of liver metastases) 4. Signed, informed consent 5. Celiac axis accessible for *bilateral* neurolysis at EUS
**Exclusion criteria**	1. Allergy to bupivacaine 2. Tumors amenable for curative resection 3. Significant coagulopathy (platelets < 50000 µ/L, INR > 1.4, uninterrupted use of oral anticoagulants at therapeutic dosage [except for antiplatelet agents] 4. Patients with concurrent sepsis 5. Previous EUS CPN

### Sample size calculations

The sample size, estimated in relation to the primary outcome, is based on the detection of a minimum clinical difference of 3 points on the Likert scale between the 2 study groups, with a beta error of 20%, an alpha error of 5%, and a standard deviation of 7. *n* = 2 × 7.8 × (7/3) 2 = 2 × 7.8 × 5.4 = 90 per group.

### Duration

Approximately 3000 EUS procedures are performed at CHUM annually, including approximately 300 adenocarcinomas of the pancreatic genu/body/tail, of which 90% (270) are inoperable and have pain. Given a sample size requirement of 90 patients and allowing for a 50% refusal/dropout rate, this study could be completed in approximately one and a half years. This was the approximate time frame for our previous RCT on neurolysis in the same patient population [4]. An extra 3 months will be necessary for completion of data analyses and drafting of the manuscript. The study will be terminated as soon as recruitment is complete. An extension will be requested if recruitment is not complete after one and a half years.

### Randomization and blinding

Participants will be randomized in 1:1 fashion, by sealed opaque envelopes, prepared by an independent third party, using a list produced by the web-based randomization service Sealed Envelope™.

Since the final inclusion criteria (accessibility of the celiac axis by EUS) is determined during the procedure, while the patient is sedated, the patient will be blinded to the treatment arm.

The research nurse who collects the data will not be present during the randomization process, nor the procedure, and will therefore also be blinded to the treatment arm.

### Study arms

1. EUS-CPN with bupivacaine: endoscopic ultrasound-guided bilateral celiac plexus neurolysis with absolute alcohol 20 mL preceded by injection of 10 ml of bupivacaine 0.5%
2. EUS-CPN: endoscopic ultrasound guided bilateral celiac plexus neurolysis with absolute alcohol 20 mL only

### Patient follow-up

**Fig. 1:**
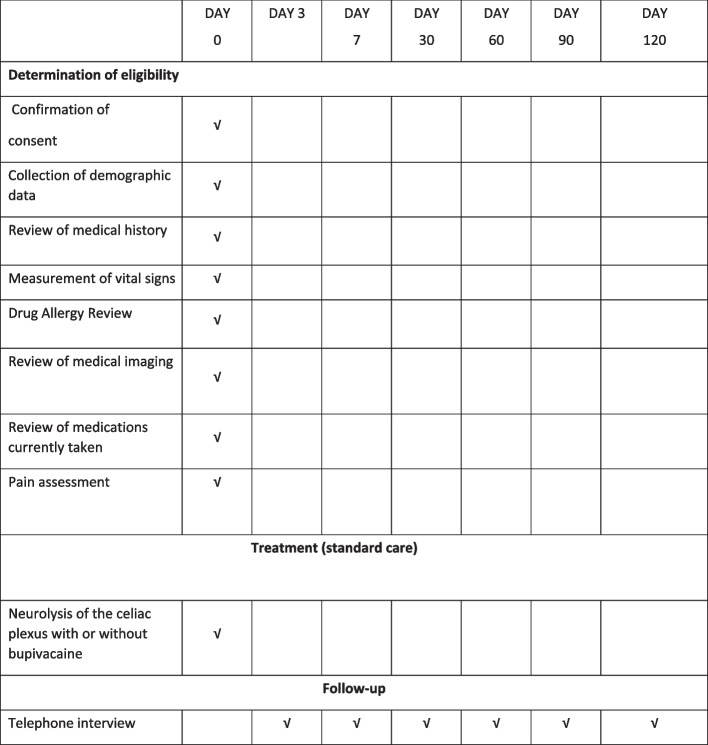
Schedule of enrolment interventions and assessments

Patients will be followed in the immediate post-procedural period until 4 months after the procedure or death whichever comes first; see Fig. 1.

Immediate post-procedural surveillance:

1. Likert pain scores pre-procedure (T0) and 30 min after arriving in recovery (T30) and then every 30 min until discharge
2. Opioid use in the last 3 days before the procedure
3. Vital signs pre-procedure (T0), immediately before and after neurolysis, and at 30 min after arriving in recovery (T30), and then every 30 min until discharge
4. Requirements for analgesia during the recovery period

Post-discharge surveillance:

Telephone interviews by a single research assistant on days 3, 7, 30, 60, 90, and 120* for:

1. 7-point Likert scale for pain (Table 2)
2. Global rating scale of change (GRSC) (Table 2)
3. Any adverse events (diarrhea, fever, dizziness, leg weakness)
4. Hospital visits
5. Narcotic use.

**Table 2:** Likert pain scale and GRSC

**7-point Likert scale for pain**	0 No discomfort at all 1 Minor discomfort 2 Mild discomfort 3 Moderate discomfort 4 Moderately severe discomfort 5 Severe discomfort 6 Very severe discomfort
**Global rating scale of change (GRSC)**	0 No improvement at all 1 Minor improvement 2 Mild improvement 3 Moderate amount of improvement 4 Quite a bit of improvement 5 A lot of improvement 6 Great improvement

*Days 3 and 7: ± 2 days; days 30, 60, 90, and 120: ± 4 days

## Outcomes

### Primary outcome

Comparative change in Likert pain scores before procedure (T0) and at 30 days after the procedure (Table 2) with treatment failure defined as less than 3-point decrease in Likert pain scores.

### Secondary outcomes

1. % Difference in 7-point Likert scale and GRSC (Table 2):
  - T0 and all other time points specified above
2. Time to discharge from the endoscopy unit
3. % Change in cumulative narcotic use for the previous 3-day period:
  - T0 vs T60 days
  - T0 vs T120 days
4. Rate of all intervention-specific adverse events (Using the American Society of Gastrointestinal Endoscopy classification) [10]
5. Survival

### Statistical analysis

Descriptive statistics will assess patient characteristics (demographics, tumor size) and the balancing of randomization. The data will be tested for normality, and the paired Wilcoxon ranked sum test will assess the pain scores. Student’s *t* test will be performed when comparing two means. Fisher’s exact method will be used for proportions comparison. All *p*-values are two-tailed, and values < 0.05 will be considered statistically significant. Subsequent logistic regression modeling will determine the factors that are associated with a better pain outcome.

Kaplan-Meier estimates of the survival curve will be obtained for each treatment group, and statistical comparisons of these will be performed using the log-rank test. The Cox proportional hazards models will be used to study survival while adjusting for potential confounding factors.

Patients who withdraw will be noted and will be noted and they will be excluded from analysis.

## Trial status

The CRCHUM Research Ethics Board (REB), more precisely its CT2 Panel, has reviewed this research project at its full board meeting where quorum was reached. It was approved, with number of 2022-9969, 21.151. Approval was communicated to the principal investigator on September 21, 2021. No REB member withdrew from the deliberations. Any protocol modification will be submitted to the REB. We have started recruitment on November 2, 2021, and 36 patients have been recruited to date. The recruitment is expected to continue until the target sample size is reached.

## Trial management

This trial was registered at ClinicalTrials.gov with registration number NCT04951804 and conducted at the CHUM only. Charles Mackay RN, who has extensive experience in clinical trials, will ensure proper trial conduct and all data collection. He will ensure proper study start-up and adaptation of the consent form. He will oversee participant enrolment, the randomization process, data collection, the timely reporting of adverse events and serious adverse events to the CRCHUM Research Ethics Board (REB), and data collection. He will ensure adherence to standard operating procedures of the CRCHUM.

## Data safety monitoring board (DSMB)

A selected group of clinicians and clinical researchers will oversee this study’s DSMB. This will include an EUS-MD from outside CHUM. Members of the DSMB will not be involved in the conduct of the trial and their participation will be void of any professional or financial bias that may hinder their independent decision making. This committee will meet before trial initiation and meet every three months to review possible AEs. Each reviewer will be blinded to the group allocation. At the first meeting, the DSMB will discuss the protocol and decide on criteria to review the data as well as monitoring of the study. The following meetings will be divided into an open and close sessions. During the open session, the PI, statistician, and all members of the DSMB will be present. The PI will present to the committee the participants’ demographic characteristics, protocol compliance, quality control, timeliness and completeness of follow-up, and all adverse events. During the closed session, the DSMB alone will review the safety data. At the end of each meeting, the members of the DSMB will vote to continue, modify, or terminate the study. The DSMB will provide a written report to the REB and PIs about their conclusion.

## Patient confidentiality

Following the signature of informed consent, participants will be given a unique identifier (ID). This study ID will be used in the main database containing study-related data. No identifying information including name, date of birth, or hospital unit number will be in the main database to protect participant confidentiality. A separate password-protected file will contain participants name and study ID information. Only the PI will have access to this file. All data will be stored for 10 years following the study completion or as requested by the standard operating procedure of the CRCHUM.

### Supplementary Information

**Additional file 1.** Copy of the case report form.

**Additional file 2.** SPIRIT checklist.

## Data Availability

The datasets used and/or analyzed during the current study are available from the corresponding author on reasonable request.
