# Supplementary material for: Protocol for a randomized controlled trial of endoscopic ultrasound guided celiac plexus neurolysis (EUS-CPN) with vs without bupivacaine
Source: Trials. 2023 Sep 8;24:576. doi: 10.1186/s13063-023-07487-7 (PMC10486028; doi:10.1186/s13063-023-07487-7)
Supplement: Supplementary file 1 — Additional file 1. Copy of the case report form. [file 13063_2023_7487_MOESM1_ESM.docx]

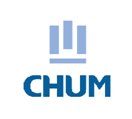

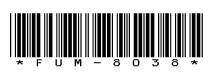


**Approved**

**–**

**CHUM**

DATE

:

6 October 2021

INITIALES

:

MJB

|  | \|  \| \| --- \| |
| --- | --- | --- |

**Informed Consent Form**

**Project title:** Randomised Controlled Trial of Endoscopic Ultrasound Guided Celiac Plexus Neurolysis (EUS-CPN) With vs Without Bupivacaine

**Responsible researcher :** D^r^ Anand Sahai, gastroentérologue, Centre Hospitalier de l’Université de Montréal (CHUM)

**Co-researcher:** D^r^ Sarto Paquin, gastroentérologue, Centre Hospitalier de

l’Université de Montréal (CHUM)

**Financial support:** None

# Number of the project at CHUM: 21.151

**________________________________________________**

NB_FICprinc_FR_V 2.0 13 août-2021

# Introduction

# We are asking for your participation in a research project because you are suffering from pain that appears to be caused by pancreatic cancer, and you need to undergo a procedure called “neurolysis of the celiac plexus” in an attempt to control this pain. However, before agreeing to participate in this project and sign this information and consent form, please take the time to read, understand and carefully consider the following information.

# This form may contain words that you do not understand. We invite you to ask any questions that you deem useful to the researcher in charge of the project or to the other members of the staff assigned to the research project and to ask them to explain any word or information that is not clear.

NATURE AND OBJECTIVES OF THE PROJECT

Often the pain caused by pancreatic cancer is treated with medicines called 'narcotics' (eg morphine). These drugs can cause side effects such as nausea, drowsiness, and constipation. Celiac plexus neurolysis is an alternative treatment that has been practiced for several years. The celiac plexus is a network of nerves located near the pancreas that is involved in transmitting pain signals from the pancreas.

Neurolysis is carried out by injecting pure alcohol around the celiac plexus, causing the destruction of the nerves transferring pain originating from the pancreas. Neurolysis can relieve pain for up to several months, which makes it possible to reduce the use of narcotics and thus reduce their adverse effects. Early application of celiac neurolysis has been shown to result in better pain control, without increased narcotics intake.

Sometimes, a local anesthetic called bupivacaine is also injected, to possibly reduce the pain that can sometimes occur when injecting pure alcohol. However, there is no data demonstrating that bupivacaine injection is effective in making the procedure less painful. Moreover, bupivacaine can even harm, diluting the therapeutic effect of alcohol.

The main purpose of this study is therefore to verify whether it is really useful to inject bupivacaine during celiac neurolysis.

Secondarily, this study will also aim to verify the improvement in quality of life associated with the reduction in the use of narcotics following neurolysis as well as to monitor survival and hospitalizations.

**NUMBER OF PARTICIPANTS AND DURATION OF PARTICIPATION**

The study will take place only at the CHUM. The total number of study participants will be 94.

The planned duration of the study will be 18 months and the duration of your individual participation will be 4 months. There will then be a follow-up until the end of life.

**NATURE OF PARTICIPATION REQUESTED**

If you agree to participate in this study, and after signing this form, you will be assigned to one of the following two groups:

**GROUP 1:** Celiac plexus neurolysis with bupivacaine

**GROUP 2:** Neurolysis of the celiac plexus without bupivacaine

Your study doctor will open a sealed envelope that will determine the group to which you will be assigned. You will have the same chance of being assigned to one or the other of the two groups. Neither you nor the research staff will know the group to which you have been assigned.

Your participation in this study will involve close follow-up by your study doctor and research staff to assess the effectiveness of the treatment, primarily through telephone interviews, as detailed in the following section.

**PROJECT PROCESS/INTERVENTIONS**

You will find a table of visits and interventions planned for the study at the end of this form.

This study will consist of 3 phases:

1) the eligibility determination period, 2) the treatment period and, 3) the follow-up period.

**1) The eligibility determination period (about 20 minutes)**

On the day of your neurolysis, you will first undergo the following assessments so that your study physician and research staff can confirm that you meet the eligibility criteria to participate in the study:

• Collection of your demographic data (age, sex and ethnicity).

• Review of medical history.

• Measurement of vital signs (blood pressure, heart rate and respiratory rate).

• Review of drug allergies.

• Review of medical imaging.

• Review of medications currently taken.

• Assessment of pain using the Likert scale (questionnaire which contains 7 answer options. The choices range from “Extremely satisfied” to “Extremely dissatisfied) and the visual analogue scale (VAS – a scale of pain self-assessment using a slider)

2) The treatment period - Standard treatment (about 2 hours)

Your study doctor will do to you a procedure called an “endoscopic ultrasound”, during which he will perform the neurolysis. The procedure lasts about 5 minutes, but your presence at the CHUM will last about 2 hours, which includes preparation time before the procedure and recovery time afterwards. Here are the steps of the intervention:

- Procedures performed as part of your standard clinical care:

• The CHUM staff will call you to set an appointment for your procedure and will give you instructions on how to prepare for it.

• Before starting the procedure, sedation (drugs creating a relaxation and drowsiness effect) will be administered intravenously to reduce the discomfort associated with the procedure and a local anesthesia of the throat will be performed using a vaporizer to control the gag reflex.

• An echo-endoscope (tube with a camera and an ultrasound probe at the end) will be introduced through the mouth into the stomach, allowing the celiac plexus to be observed.

• A verification of the accessibility to the celiac plexus will be carried out. If this is inaccessible, neurolysis will not be possible and you will not be able to continue to participate in the study.

• Neurolysis will then be carried out using a needle introduced into the echo-endoscope and then directed through the wall of the stomach to the celiac plexus. Pure alcohol will then be injected into the celiac plexus.

• Once the procedure is over, you will have a recovery period of approximately one hour. When the effect of the sedation will be almost completely dissipated, the return home will be authorized, preferably in the company of a loved one. You will be prohibited from driving a vehicle until the following day.

**Procedures carried out for research purposes:**

• During the procedure, if accessibility to the celiac plexus is confirmed, your study doctor will open a sealed envelope which will assign you to one or the other of the two study groups.

• The injection of pure alcohol may or may not be preceded by an injection of bupivacaine, depending on the group to which you have been assigned.

• During your recovery period, the research staff will collect certain data (pain levels, vital signs, length of recovery period).

• A post-intervention guide will be given to you before your departure.

**3) The follow-up period (expected duration of each call: 10 minutes)**

The research staff will contact you by telephone on days 3, 7, 30, 60, 90 and 120 following neurolysis, in order to follow up on the following points:

• Assessment of pain using the Likert scale (questionnaire which contains 7 answer options. The choices range from “Extremely satisfied” to “Extremely dissatisfied) and the visual analogue scale (VAS – a scale of pain self-assessment using a slider)

• Evaluation of the improvement in your state of health using a scale

• Evaluation of the presence or absence of side effects related to neurolysis

• Hospitalization(s) since neurolysis, if applicable

• Review of the use of narcotic drugs to treat pain.

In the event that it is not possible to communicate with you to carry out this follow-up, and if you consent to it at the end of this form, the researcher in charge of this research project or a member of his staff may contact your attending physician. or the identified person(s) designated by you to obtain information on your general state of health or the date of your death if this occurs during the study period. The research team could also attempt to obtain information from public records, if available.

**YOUR RESPONSIBILITIES AND IMPORTANT PRECAUTIONS TO TAKE**

• You must notify research staff if you are allergic to bupivacaine.

• By signing this consent form, you agree to follow the instructions of your study doctor, to attend scheduled visits in connection with the study and to undergo all assessments required as part of the study. study.

• You should tell your study doctor or a member of their team as soon as possible if you experience any unusual symptoms or side effects, as this could affect your health. You can reach them at the telephone number indicated in the “Identification of resource persons” section.

In the event of an emergency (evenings, nights, weekends and holidays), to report side effects or any research-related injury, you must go to the CHUM emergency room if necessary and you will be seen. by the doctor on duty in gastroenterology. You must mention that you are participating in this research project.

**RISKS AND INCONVENIENCE –**

**Risks Associated with Celiac Plexus Neurolysis**

Since neurolysis of the celiac plexus is a standard care intervention that was already included in your medical treatment plan, no risk will arise from your participation in this research project.

**- Risks associated with bupivacaine:**

Administration of bupivacaine may cause the following rare side effect (less than 1%):

• Allergic reaction, in the form of a skin rash, which would appear immediately after the procedure.

There is also a theoretical risk of accidentally injecting bupivacaine into a blood vessel. This would immediately cause an arrhythmia (irregular heartbeat) followed by a significant drop in blood pressure that could lead to death. No accidental injections have been documented so far.

Note that your study doctor does not anticipate a significant increase in pain in the absence of bupivacaine.

**- Disadvantage related to the participation time**

The time spent participating in the study, as described above, could be an inconvenience.

**ADVANTAGES**

You may derive personal benefit from your participation in this research project, but this cannot be guaranteed. At the very least, the results obtained will contribute to the advancement of knowledge in this field.

**PRIVACY**

During your participation in this research project, the researcher in charge of this project as well as the research team will collect, in a research file, the information concerning you and necessary to meet the scientific objectives of the research project.

This information may include information contained in your medical file concerning your past and present state of health, your lifestyle habits as well as the results of all tests, examinations and procedures that will be carried out. Your record may also include other information such as your name, gender, date of birth and ethnicity.

All data collected (including personal information) will remain confidential within the limits provided by law. You will only be identified by a code number. The code key linking your name to your research file will be kept by the researcher responsible for this research project.

To ensure your safety, a copy of the consent form will be placed in your medical file. Therefore, any person or company to whom you give access to your medical file will have access to this information.

This research data will be kept for at least 25 years after the end of the study by the researcher responsible for this research project.

Research data may be published or discussed scientifically but will not identify you.

For the purposes of monitoring, control, protection and safety of the intervention under study by regulatory bodies, your research file as well as your medical file may be consulted by a person mandated by regulatory bodies, in Canada. or abroad, such as Health Canada, as well as by representatives of the institution or the research ethics committee. These people and organizations will have access to your personal data, but they adhere to a privacy policy.

You have the right to consult your research file to verify the information collected and have it corrected if necessary.

**COMMUNICATION OF GENERAL RESULTS**

You will be able to know the general results of this study if you ask the responsible researcher at the end of the study.

**POSSIBILITY OF MARKETING**

The research results resulting from your participation in this project, among other things, could lead to the creation of commercial products. However, you will not be able to derive any financial benefit from it.

**PROJECT FINANCING**

The researcher responsible for the project and the institution did not receive any funding to carry out this research project.

**COMPENSATION** See Appendix - Tables ex

You will not receive financial compensation for your participation in this research project.

**IN CASE OF DAMAGE** See Appendix -

If you should suffer any harm whatsoever as a result of any procedure related to this research project, you will receive all the care and services required by your state of health.

By agreeing to participate in this research project, you do not waive any of your rights and you do not release the researcher responsible for this research project and the establishment from their civil and professional liability.

**VOLUNTARY PARTICIPATION AND RIGHT OF WITHDRAWAL**

Your participation in this research project is voluntary. You are therefore free to refuse to participate. You can also withdraw from this project at any time, without having to give reasons, by informing the researcher responsible for the research project or a member of the research team.

Your decision not to participate in this research project or to withdraw from it will have no impact on the quality of care and services to which you are entitled or on your relationship with the teams providing them.

The researcher responsible for this research project or the research ethics committee may end your participation without your consent. This can happen if new discoveries or information indicate that your participation in the project is no longer in your interest, if you do not respect the instructions of the research project or if there are administrative reasons to abandon the project.

If you withdraw from the research project or are removed from the project, no further data will be collected and no further samples will be taken. The information will nevertheless be retained, analyzed or used to ensure the integrity of the research project, as specified in this document.

Any new knowledge acquired during the course of the research project that could have an effect on your decision to continue to participate in it will be communicated to you quickly.

**SECONDARY USE OF DATA**

The Lead Investigator may use and share study data/information with other research partners or academic researchers to answer questions not directly related to this study but that would advance science, medicine, and public health. Appropriate measures will also be put in place to prevent unintended re-identification of the identifying information of the patients whose records were the subject of this study.

The potential use of your data includes the following examples:

• Independent confirmation of the results of this study;

• Advancement of knowledge on the use of bupivacaine in the context of neurolysis of the celiac plexus and on the associated health problems;

• Planning of possible study projects;

• Development of various uses and reviews of scientific data;

• Development of and access to new drugs, medical devices, and healthcare solutions.

The results arising from your research data may be published or be the subject of scientific presentations by the individuals or organizations that receive your data; your data may also be used for educational purposes. Please note that the Research Ethics Board and the research team do not have access to details regarding the possible use of your research data. This secondary use could be limited to what has been described in the consent form. If other uses not described in this form (including in this section) were planned, the sponsor of this study undertakes to inform the ethics committee before proceeding.

**OTHER POSSIBLE TREATMENTS**

If you decide not to participate in this study, your doctor will discuss other available treatments with you.

**IDENTIFICATION OF RESOURCE PERSONS**

If you have any questions or experience problems in connection with the research project, or if you wish to withdraw from it, you can communicate with the researcher in charge, Dr. Anand Sahai, or with a person from the research team at the following number : 514-890-8000, ext. 36484, between 8 a.m. and 4 p.m., Monday to Friday.

If you have any questions regarding your rights as a participant in this research project or if you have any complaints or comments to make, you can contact the CHUM local service quality and complaints commissioner at 514- 890-8484.

**INFORMATION ABOUT THE STUDY ON A WEBSITE**

A description of this study is available in English only at http://www.ClinicalTrials.gov. This site does not contain any information that identifies you. The site will include at most a summary of the results when they are available. You can consult this site at any time. The registration number for this project is NCT04951804.

**APPROVAL BY THE RESEARCH ETHICS BOARD**

The CHUM Research Ethics Committee has given its approval and will monitor it.

**SIGNATURE**

I have read the information and consent form. I was told about the research project and this information and consent form. My questions were answered and I was given enough time to make a decision. After reflection, I agree to participate in this research project under the conditions set out therein.

The type of project in which I am asked to participate generally requires, following the active part of the treatment, a follow-up throughout my life. On the other hand, it happens that over time, we lose contact with certain participants. To ensure the validity of the research, it is important to know the date and the cause, in the event of death. Signing this consent form authorizes the Ministry of Health and Social Services to communicate this personal information to the researcher, when necessary.

I authorize the research team to have access to my medical file.

I also agree that the research team may continue to have access to my medical file and to use my information or samples if I lose my decision-making capacity, or after my death, for the sole purposes of the research objectives, within the framework of this project.

I agree that the person whose contact details are given below may be contacted by my study doctor or a member of his research team, to follow up on my state of health if they are unable to join:

Yes

No

Name and contact details of the contact person:

Name Signature of the participant Date

**SIGNATURE OF PERSON WHO OBTAINS CONSENT**

I explained to the participant; the research project and this information and consent form and I answered the questions he/she asked me.

Name Signature of the person obtaining the consent Date

**COMMITMENT OF THE RESPONSIBLE RESEARCHER AT THE CHUM**

I certify that the present information and consent form has been explained to the participant, that the questions that the research subject had have been answered.

I agree, with the research team, to respect what was agreed in the information and consent form and to give a signed and dated copy to the participant.

**SIGNATURE OF WITNESS**

**YES □ NO □**

The signature of a witness is required for the following reasons:

Difficulty or inability to read - The person (impartial witness) who affixes his/her signature below attests that the consent form has been read and that the project has been explained precisely to the participant, who seems to have understood it.

Misunderstanding the language of the consent form - The person signing below acted as an interpreter for the participant during the consent process.

Name Signature of the witness Date

**Please note:**

**Further details of assistance provided during the consent process should be documented in the participant's research record, if applicable.**

Table of study visits and interventions

|  | DAY  0 | DAY 3 | DAY  7 | DAY  30 | DAY  60 | DAY  90 | DAY  120 |
| --- | --- | --- | --- | --- | --- | --- | --- |
| **Determination of eligibility** | |  | | |  |  |  |
| Confirmation of  consent | √ |  |  |  |  |  |  |
| Collection of demographic data | √ |  |  |  |  |  |  |
| Review of medical history | √ |  |  |  |  |  |  |
| Measurement of vital signs | √ |  |  |  |  |  |  |
| Drug Allergy Review | √ |  |  |  |  |  |  |
| Review of medical imaging | √ |  |  |  |  |  |  |
| Review of medications currently taken | √ |  |  |  |  |  |  |
| Pain assessment | √ |  |  |  |  |  |  |
|  | | **Treatment (standard care)** | | |  |  |  |
| Neurolysis of the celiac plexus with or without bupivacaine | √ |  |  |  |  |  |  |
|  | | **Follow-up** | | |  |  |  |
| Telephone interview |  | √ | √ | √ | √ | √ | √ |

Page
